# Supplementary material for: Role of RPA Phosphorylation in the ATR-Dependent G2 Cell Cycle Checkpoint
Source: Genes (Basel). 2023 Dec 13;14(12):2205. doi: 10.3390/genes14122205 (PMC10742774; doi:10.3390/genes14122205)
Supplement: Supplementary file 1 [file genes-14-02205-s001.zip › genes-2761219-supplementary.pdf]

# Role of RPA phosphorylation in the ATR-dependent G2 cell cycle checkpoint

Shengqin Liu, Brendan M. Byrne, Thomas N. Byrne and Greg G. Oakley

Supplemental Figure 1

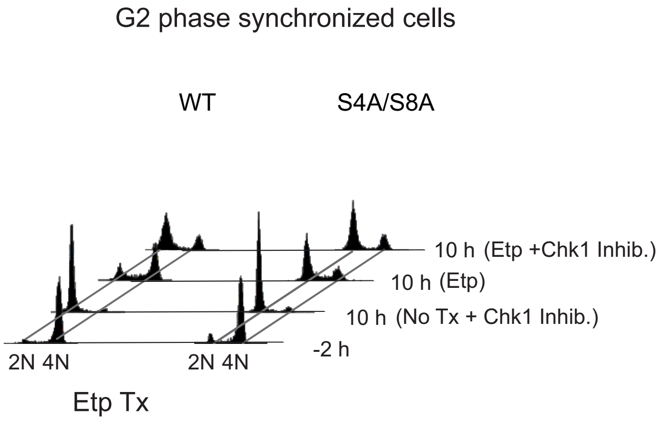


Supplemental Figure 1. FACS analysis of cells following synchronization in G2. Comparison of WT and S4A/S8A RPA32 expressing cells treated as indicated for 2 h with etoposide (20 μM) and/or Chk1 inhibitor SB218078 and analyzed at 10 h after etoposide removal.
